# Adolescent Engagement With a Multicomponent mHealth Tool: Identifying Usage Patterns, Determinants, and Health Behavior Change in an Intervention Trial

**DOI:** 10.2196/59041

**Published:** 2025-08-18

**Authors:** Carmen Peuters, Ann DeSmet, Laura Maenhout, Greet Cardon, Dries Debeer, Geert Crombez

**Affiliations:** 1 Department of Experimental-Clinical and Health Psychology, Faculty of Psychology and Educational Sciences, Ghent University, Henri Dunantlaan 2, Ghent, 9000, Belgium, 32 9 264 64 61; 2 Department of Movement and Sports Sciences, Faculty of Medicine and Health Sciences, Ghent University, Ghent, Belgium; 3 ISGlobal, Barcelona Institute for Global Health - Campus MAR, Barcelona Biomedical Research Park (PRBB), Carrer del Doctor Aiguader, 88Barcelona, 08003, Spain, 0034 932 14 73 49; 4 Faculty of Psychology, Educational Sciences and Speech Therapy, Université Libre de Bruxelles, Brussels, Belgium; 5 Department of Communication Studies, University of Antwerp, Antwerp, Belgium; 6 Department of Experimental Psychology, Faculty of Psychology and Educational Sciences, Ghent University, Ghent, Belgium

**Keywords:** behavioral engagement, experiential engagement, multicomponent interventions, unstructured apps, cluster analysis, mobile health, behavior change, system log data, law of attrition, tailoring digital health interventions, teens, youth, adolescents, engagement, support chatbot, gamification, activity tracker, logistic regression, sleep quality, mobile phone

## Abstract

**Background:**

Research about the engagement of adolescents with mobile health (mHealth) interventions is scarce, while it is generally assumed that the engagement affects the intervention efficacy.

**Objective:**

Using an mHealth intervention that targets the general population of adolescents to promote healthy behaviors (physical activity, low sedentary time, adequate sleep, and taking breakfast) and mental health, we aimed to investigate (1) how adolescents engage with the intervention, (2) which engagement styles can be identified and how these differ according to personal characteristics, and (3) which style of engagement predicts behavior change. The intervention used, #LIFEGOALS, includes self-regulation techniques, a support chatbot, narrative videos, and gamification, brought together in an app coupled to an activity tracker.

**Methods:**

Logged usage data and self-reports of experience with #LIFEGOALS were collected from 159 adolescents (mean age 13.54, SD 0.95 years) over a 12-week intervention period and used to describe behavioral and experiential engagement with the intervention components over time. Baseline data on sociodemographic variables, mental health, and behavioral determinants were explored as determinants of engagement and were used to characterize engagement styles that were identified through exploratory cluster analysis on the frequency of usage of the components. Linear mixed-effects regression was used to analyze the effect of engagement style on health behavior change.

**Results:**

Average time in the app was 26 minutes (SD 26) over the 12-week period, with usage decreasing substantially after the first week. The use of self-regulation techniques and gamification was strongly interrelated (0.65 <*r* <0.70), whereas use of Fitbit showed weaker correlations with other component usage (0.15 <*r* <0.31). Exploratory analyses suggest that engagement was influenced by immigration background and by adolescents’ attitudes, self-efficacy, and intentions toward healthy living. Younger participants tended to use the Fitbit more frequently. Cluster analysis identified 4 engagement styles: narrative usage (n=19), app usage (n=36), Fitbit usage (n=32), and no usage (n=72), which were associated with differences in age, peer support, and mental health. Engagement style did not affect change in health behavior outcomes from preintervention to postintervention.

**Conclusions:**

Different engagement styles were identified based on the frequency and type of components used. Findings support the relevance of tailoring mHealth to individual, interpersonal, and contextual characteristics. The overall low engagement with the intervention may have limited the detection of differences in health effects between engagement styles.

## Introduction

Mobile health (mHealth) is defined as the use of mobile devices such as smartphones and activity trackers in health care. Compared with in-person care, mHealth may offer advantages in promoting health by reducing barriers such as cost, stigma, confidentiality concerns, and scheduling or distance constraints [1,2]. mHealth may also facilitate health empowerment by providing personalized, real-time information and support [3] and by enabling independent health management that can bypass parental dependence [2,4]. Health apps may be particularly suitable for adolescents [5]. Following statistics from Western countries, between 93% and 98% of teens aged 12-17 years own a smartphone [6-8]. In Flanders, Belgium, 96% of adolescents use their smartphone more than an hour, and 45% even more than 4 hours on days without school [9]. The integration of smartphones in adolescents’ daily life may facilitate the adoption of a health app. Nevertheless, it remains a challenge to get adolescents to initiate and sustain engagement with digital health interventions. In research trials, a high percentage of adolescents do not use the app or drop out early [10-13]. These data are estimated to be even higher in real-world usage [14]. Low intervention usage relates to fewer positive health effects [15-17], but little is known about the quality of engagement and how this relates to health outcomes.

Intervention log analytics is an approach to study and interpret engagement with digital interventions. For unstructured apps (ie, consisting of variable components that users can access and use at will), useful indicators are the frequency of interactions logged, the number of app features accessed, the number of activities completed, and the number of minutes spent in a web-based program [18,19]. Logged usage data have been used to determine user styles, either via cluster analysis or by transforming a continuous engagement outcome into discrete categories [16,18]. Users are mainly classified according to the extent of usage of the intervention [15-17,20], but user patterns can also be explored in terms of engagement with different intervention components or features. This might be relevant for multicomponent and (partly) unstructured interventions where it can reveal naturalistic user patterns, such as preferences for (the combination of) certain intervention features and their relationship with intervention effects. A few studies have identified clusters that reflect a difference in the use of different components [17,20]. Besides a cluster with overall low usage, the remaining clusters distinguished between using mainly 1 component of the intervention and using that component in combination with other features of the intervention. Effects of these types of clusters on health outcomes are mixed and likely depend on the intervention content and components. For example, the analysis of engagement with a digital intervention for young adults recovering from first-episode psychosis found health improvements only for the group with maintained use of the therapy and the social component but not for the group with maintained use of only the social component [17]. Another web-based intervention for adults with mild to moderate depression, anxiety, and stress did not find an effect of the clusters on health outcomes [18].

With the aim of maximizing intervention effects, an identification of different engagement styles and their health effects should be complemented with an understanding of the factors that influence patterns of engagement. Intervention characteristics (eg, novelty, autonomy, or complexity) or intervention components (eg, reminders or social support features) can have a different impact on individuals’ engagement depending on user characteristics (eg, motivation for the health behavior, mood, or education level) [21]. More research is needed on the determinants of different aspects of engagement to inform future design and tailoring of effective mHealth interventions.

This study uses the multicomponent mHealth intervention #LIFEGOALS as a case study to (1) describe engagement with the intervention, (2) identify and characterize clusters of engagement, and (3) analyze their influence on health behavior change. #LIFEGOALS is an app coupled with an activity tracker to guide adolescents toward healthier lifestyles for promoting mental health. The intervention showed beneficial effects on physical activity, sedentary behavior, sleep quality, and emotions (ie, in a close-to-normal situation, not in case of COVID-19 pandemic restrictions) [22].

## Methods

### Study Design

This study used data from adolescents who participated in a quasi-randomized controlled effect study of the #LIFEGOALS intervention [22]. Participants were recruited from the general population of adolescents enrolled in nonspecial needs secondary education in Flanders, Belgium. The recruitment process was designed to ensure adequate representation of students from the vocational education track, which focuses on practical and job-oriented training. In Flanders, adolescents in the vocational track typically come from lower socioeconomic backgrounds compared with their peers in the general or technical tracks [13] and are at greater risk for engaging in unhealthy behaviors [23]. From the 184 participants allocated to the intervention group, 25 could not install the app due to technical reasons and were not included in this study, leaving a final sample of 159 adolescents (mean age 13.54, SD 0.95 years; 82/159, 51.6% girls). Data were collected in 3 waves. All waves included 2 intervention schools and started baseline assessment in October 2020, November 2020, and January 2021, respectively. At baseline, and after 2, 6, and 12 weeks of intervention, participants completed a web-based survey. Following the baseline assessment, participants were assisted to install the #LIFEGOALS app on their smartphone and were given a Fitbit Charge 2 or 3 on-lend to link with the app. The intervention was introduced as a health app to improve lifestyle, and participants were invited to use the intervention in the next 12 weeks. As it concerns an unstructured app, users could access all components at all times and could engage with the intervention as they wished. The working of the app was explained in the classroom by means of a slide show. In the last wave of data collection (n=62), the first episode of the narrative was shown in the classroom because low viewership numbers for the narrative were observed in waves 1 and 2.

### Intervention

#LIFEGOALS is an mHealth promotion multicomponent intervention for Flemish adolescent youth, which aims to promote mental health by improving healthy lifestyle behaviors. The app focuses on increasing physical activity, reducing sedentary behavior, improving adequate sleep, and daily breakfast consumption. The intervention is theory-based (Health Action Process Approach [24], Persuasive Systems Design [25], and extended Elaboration Likelihood Model [26]) and was developed using a participatory design approach. It includes (1) self-regulation techniques guiding users through goal setting, action and coping planning, and self-monitoring (using an activity tracker and graphs) to help bridge the intention-behavior gap; (2) a health narrative in the format of an entertaining storyline offered in weekly short video episodes to facilitate health behavior change through identification with the characters; (3) an automated, rule-based (non–artificial intelligence) chatbot for individual support by providing information and sending supporting messages; and (4) other content and features such as health information and gamification. The use of the #LIFEGOALS intervention was stimulated by placing a roll-up banner in the participating classes to remind participants of using the app. The intervention is described in more detail elsewhere [27], and the home screen is illustrated in Figure 1.

**Figure 1.**
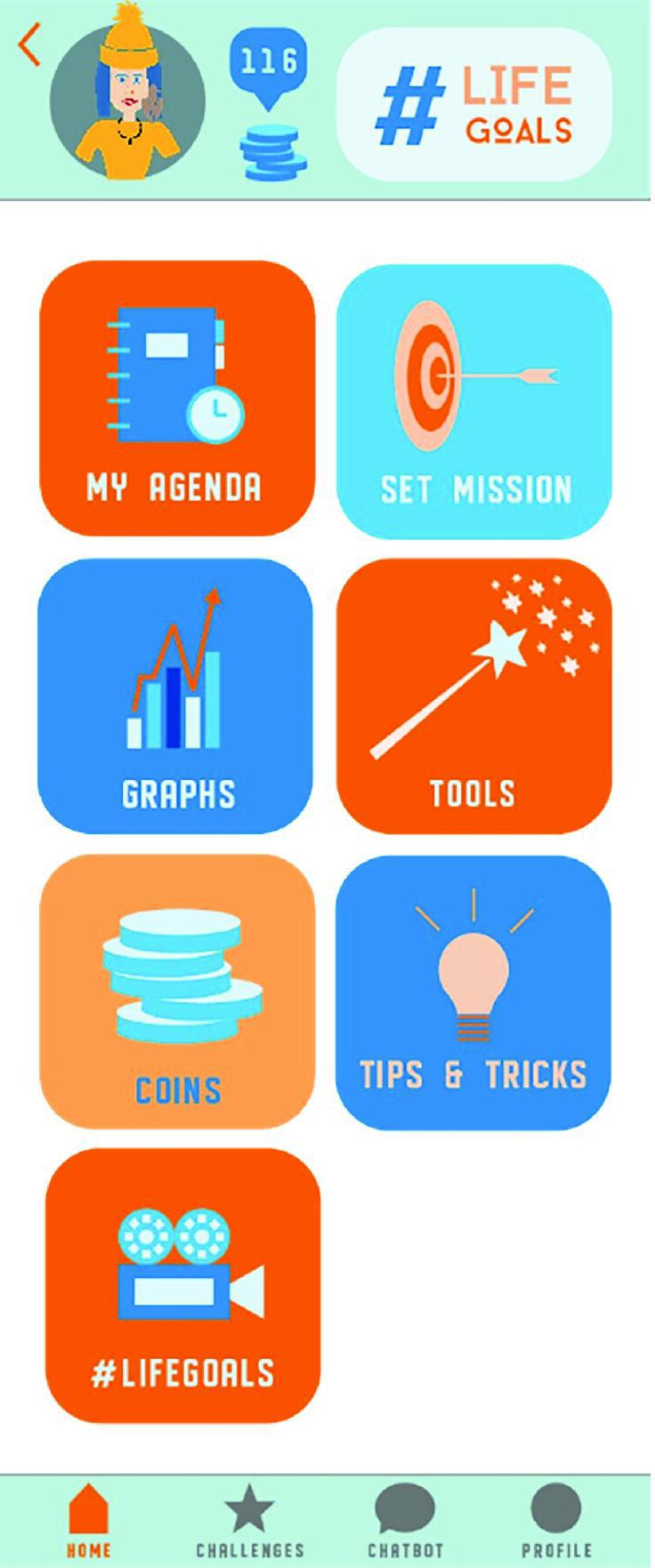
Home screen of the #LIFEGOALS app.

### Ethical Considerations

The study was approved by the ethics committee of the Faculty of Psychology and Educational Sciences of Ghent University (registration no.: 2020/70). Participants, their parent or guardian, and the school director received an information form tailored to their role detailing the study process, data protection measures, and the right of the participants to withdraw. Written consent was obtained before participation. During data collection, participants’ identities were safeguarded through pseudonymization, using an encrypted key file to link participant codes with identities. After data collection and merging of user log data, questionnaire follow-ups, and baseline data, the dataset was fully anonymized and the key file was permanently destroyed. As an incentive, participants received a power bank after the baseline assessment and a cinema ticket upon completing the follow-up measurement.

### Measures

#### Behavioral Engagement

By means of a unique identifier that had to be entered when installing the #LIFEGOALS app, user app activity was continuously logged throughout the 12-week intervention period. Log files included a date stamp and time stamp, the sort of activity (eg, filling out a certain section of the app, setting a certain choice or content, or receiving a push message by the app), and the content of the activity, if relevant (eg, text filled in by the participant, the option that was chosen, or the content of the push message). The usage of different components was operationalized by the frequency of opening each component section in the app (Table 1). Usage of the narrative was additionally operationalized by the self-reported number of watched episodes after week 1 (answer options: 0, 1, or all 2), after week 6 (0, 1 or 2, or 3 or more), and after week 12 (0, 1 or 2, 3-11, or all 12). Likewise, for the Fitbit, a unique login provided during installation gave the researchers access to the Fitbit data after participants synchronized their Fitbit in the Fitbit app. A total number of daily steps that exceeded zero was coded as a day with Fitbit usage.

**Table 1.** The different app components.

App component	Description
Action planning	Self-regulation technique. Via the button “Set mission” users are guided to translate a goal (eg, increase physical activity) into an action plan (eg, going to school by bike on Friday at 8 AM).
Coping planning	Self-regulation technique. When creating their action plan, users are asked to think of possible obstacles that may occur (eg, forgetting) and to think ahead of possible solutions for that obstacle (eg, hang post-its). Coping planning is, furthermore, accessible via the “Tools” button.
Agenda	The button “My agenda” offers a week overview of all planned missions, as well as a list “My missions” where—for every mission in the past—users can tick off whether they managed to fulfill the mission.
Chatbot	“Botty” is a chatbot to which users can ask questions (about a healthy lifestyle or the functioning of the app) and which sends encouraging messages and an announcement when a new episode of the narrative is available.
Narrative	Twelve short video episodes (3-6 minutes each) screen the everyday life of 4 adolescent classmates with a focus on entertainment. Implicitly, the storyline shows the importance of a healthy lifestyle for mental well-being. Each week, a new episode comes available in the app.
Gamification	Users can earn “coins” by planning and completing missions. Earned coins can be used to purchase attributes to personalize an avatar. Gamification usage was operationalized by summing the frequency of opening the app sections “Coins” and “Avatar.”
Progress graphs	The “Graphs” button presents graphical representations of user progress to self-monitor their behavior (number of steps, minutes spent in physical activities, sleep duration, and frequency of taking breakfast).
Information	Under the “Tips & Tricks” button are included: information about the health benefits of the targeted behaviors, inspiration on how to perform the health behaviors, and referral to other organizations and websites for more information or guidance.

#### Experiential Engagement

After week 1, week 6, and postintervention, participants reported whether they had watched any episode of the narrative and whether they had used the other parts of the app. Participants who reported nonusage were asked in an open question for the reason. For participants who indicated at least minimal usage, their user experience was measured for, respectively, the narrative component and the other parts of the app. Items from the Digital Behavior Change Intervention Engagement Scale [28] were translated and specified to the #LIFEGOALS narrative or the other parts of the app. After factor analysis on data of the pilot study (n=35), items were limited to 3 (interest, attention, and affect) for *experiential engagement with the narrative*, and 4 (interest, attention, affect, and usefulness) for *experiential engagement with the other parts of the app*. Answers on a 5-point Likert scale (totally disagree—totally agree) were averaged with higher scores indicating higher experiential engagement.

#### Behavioral Determinants

*Attitude* (ie, the tendency to evaluate behavior with some degree of favor or disfavor), *self-efficacy* (ie, the belief in one’s capability to perform the behavior), and *intention* (ie, the explicit decision to act in a certain way) were assessed separately for physical activity, sedentary behavior, sleep, and frequency of breakfast consumption. The items were based on scales used in other studies ([29] for physical activity and sedentary behavior; [30] for sleep) from which the highest loading items from factor analysis were selected (retaining 3 items for attitude, 3 for self-efficacy, and 1 for intention). The scores from 1 (not agree) to 5 (totally agree) for the different behaviors were averaged to reflect the determinant toward a healthy lifestyle in general.

#### Health Behaviors and Mental Health

*Physical activity*, *sedentary behavior,* and *sleep time* were measured at baseline and postintervention using Axivity AX3 accelerometers worn on the nondominant wrist for 7 consecutive days. Raw accelerometry data were processed in R to obtain the average Euclidean Norm Minus One per minute (ie, volume of physical activity) expressed in milligravity-based acceleration units (mg), the average of sedentary minutes per day (ie, sedentary time), and weekday-to-weekend sleep time difference (ie, sleep regularity) (see the study by Peuters et al [22]). Phillips cut points [31] were used to classify each activity minute into light or moderate to vigorous intensity physical activity. *Sleep quality* (ie, a composite score of items on sleep latency, sleep interruption, daytime sleepiness, and experience of sleep quality, based on the studies by Inchley et al [32] and Essner et al [33]) and *frequency of breakfast consumption* (ie, the average days per week taking breakfast [32]) were assessed in the web-based survey at baseline and postintervention.

*Health-related quality of life, psychological well-being, emotions, self-perception,* and *peer support* were assessed using the KIDSCREEN-10 [34] and subscales from the KIDSCREEN-27 [35] and KIDSCREEN-52 [36]. Higher *t*-scores (mean 50, SD 10) indicate better mental health. *Resilience* was measured with an adapted Dutch version of the Brief Resilience Scale [37], with higher averaged scores indicative of greater resilience. *Depressed feelings* were measured using the Custom Short Form of the Dutch version of the PROMIS-PedDepSx (Patient-Reported Outcomes Measurement Information System Pediatric Bank Depressive Symptoms v2.0) [38], with higher *t*-scores (mean 50, SD 10) indicating more depressed feelings.

#### Pandemic-Related Education Measures

The data collection took place during the second wave of the COVID-19 pandemic in Belgium. In this period in Belgium, people were allowed to leave the house by foot or bike, but other governmental measures were implemented to limit the spread of infections. One of these measures that might have affected adolescent usage of the intervention was a minimum of 50% per week distance learning for adolescents in the third year of secondary education and above (not affecting first and second years). A binary variable was created representing the presence of education restrictions (normal in-school education vs partly remote education).

### Statistical Analyses

#### Describing Engagement

Linear mixed models with school class as random intercept (to deal with the nested nature of the data) were used to analyze differences in engagement according to individual characteristics. Frequency variables were log-transformed to account for their skewed distribution. The reported statistical tests use Satterthwaite’s method to approximate degrees of freedom, as implemented in the *lmerTest* R package [39]. Given the exploratory nature of these analyses including multiple comparisons, *P* values are reported descriptively and not interpreted for significance.

#### Identifying Engagement Styles

The *cluster* R package [40] and R (version 4.0.5) were used to identify clusters in the engagement data. The selection of which engagement indicators to include in the final cluster analysis was based on the descriptive statistics of engagement and on the theoretical and evidence-based rationale for inclusion of the different components in the intervention (Multimedia Appendix 1). Because data of mixed types (continuous for frequency and ordinal for narrative episodes) were used, Gower distance [41] was used as the distance measure. The frequency variables were right-skewed and, therefore, log-transformed. Partitioning Around Medoids was used as an iterative clustering procedure. Average Silhouette Width was used to decide on the optimal number of clusters. Only those solutions with 3 or more clusters (to explore patterns of engagement outside of a dichotomous high vs low use range) and only those solutions where all clusters consisted of ≥15 participants (to be able to perform follow-up analyses) were considered.

#### Determinants of Engagement Style

Differences between clusters on baseline characteristics were investigated with multinomial logistic regression using the *nnet* R package [42]. Given the nested nature of the data (ie, students nested within classroom), we ran multilevel versions of the models with school class as random intercept. However, these models did not explain additional variance and resulted in the same conclusions, for which only the results from the models without multilevel structure are presented. Due to the higher number of missing values in the accelerometry data, a separate model was fitted for the health behaviors versus the sociodemographic variables (age, gender, and family affluence), behavioral determinants (attitude, self-efficacy, and intention), and mental health indicators. Education restrictions (ie, the pandemic-related measure of half-time remote education) were included as a covariate because they might have influenced engagement with the intervention by reducing live peer interactions. The results of the full models are added in Multimedia Appendix 2. Variables that were significantly associated with the clusters (ie, age, intention, self-perception, peer support, depressed feelings, and education restrictions) were standardized and included in the final analysis.

#### Predicting Health Outcomes Based on Engagement Style

For each behavioral outcome, the *lmerTest* R package [39] was used to fit a linear mixed model with random intercepts for participant and school class to test the interaction between time (preintervention vs postintervention) and cluster. For the analysis of physical activity, 1 outlier was detected by inspecting residual plots and was left out of the analysis.

## Results

### Participant Characteristics

Sample characteristics are shown in Table 2. The participants were evenly divided over the first, second, and third year of secondary education. The proportion of participants following the vocational education track was comparable with the 24.5% of the overall Flemish population in 2020‐2021 [43]. Distribution of family affluence into low, middle, and high affluence was comparable with the general Flemish population [32]. See the study by Peuters et al [22] for sample characteristics of mental health and lifestyle behaviors.

**Table 2.** Sample descriptives (N=159).

Characteristics	Values, n (%)
Gender	
Girl	82 (51.6)
Boy	75 (47.2)
Other	2 (1.3)
Education type	
General or technical	120 (75.5)
Vocational	39 (24.5)
Grade	
First year	51 (32.1)
Second year	56 (35.2)
Third year	52 (32.7)
Family affluence	
Low	47 (29.6)
Middle	77 (48.4)
High	35 (22.0)
Language at home	
Dutch	129 (81.1)
Other	30 (18.97)

### Describing Engagement

#### Usage Over Time

The components that were used the most were the action and coping planning and the Fitbit, followed by the gamification and the chatbot. Users visited the narrative section in the app only a few times, but it should be taken into account that weekly only 1 new episode of the narrative appeared in the app. Behavioral engagement with the app and Fitbit drastically decreased after the first week (see Multimedia Appendix 3 for boxplots of the usage frequency of the different intervention components). Of the participants who installed the app, 76.1% (121/159) never opened the app in the last half of the intervention period. Continued usage with the Fitbit was also low, with 42.8% (68/159) of the participants who never synchronized the Fitbit with the Fitbit app in the second half of the intervention. Experiential engagement was more stable over time (Multimedia Appendix 3). Average experiential engagement at the end of the first week was positive with the app (mean 3.55, SD 0.71) and with the narrative component (mean 3.68, SD 0.68) and dropped slightly by the end of weeks 6 and 12. Of the participants who completed the postsurvey, 25.8% (38/147) indicated that they had not really used the app. The most common reasons for not or no longer using the app after week 1, week 6, or week 12 are shown in Table 3, together with the number of participants reporting this reason.

**Table 3.** Participant reasons for low usage of the #LIFEGOALS app after week 1, week 6, and week 12.

Reason for nonusage	Week 1^a^, n (%)	Week 6^b^, n (%)	Week 12^c^, n (%)
Low ease of use (app was unclear or complicated to use)	4 (20.0)	3 (15.8)	7 (18.4)
Lack of time (having no time or too little time to use the app)	3 (15.0)	7 (36.8)	4 (10.5)
Forgotten to use the app	5 (25.0)	2 (10.5)	6 (15.8)
Technical problems (crashes, slow, or not being able to install)	4 (20.0)	1 (5.3)	6 (15.8)
No interest or not useful (no motivation, no need to use the app)	1 (5.0)	3 (15.8)	6 (15.8)
App unattractive (was no fun or boring)	0 (0.0)	0 (0.0)	3 (7.9)
Fitbit app was easier to use than the #LIFEGOALS app	1 (5.0)	0 (0.0)	1 (2.6)
Other (eg, did not know why)	2 (10.0)	3 (15.8)	7 (18.4)

a n=20; missing=14.5% (23/159).

b n=19; missing=23.9% (38/159).

c n=38; missing=7.5% (12/159).

#### Associations Between Intervention Components

**Figure 2.**
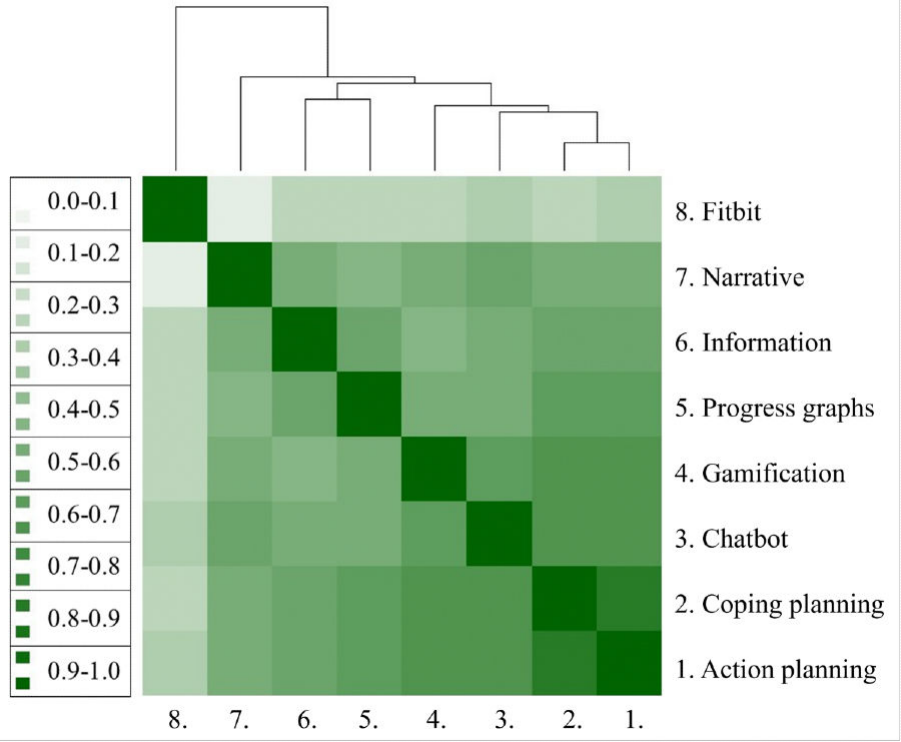
App component associations. Heat map (rank-order correlations) and dendrogram of the frequency of usage of the different app components. Darker colors represent stronger positive associations.

Interdependence between the intervention components was calculated via Spearman rank order correlations between the frequencies of usage (Multimedia Appendix 3), and is shown in an association heat map (Figure 2). The frequency with which the action and coping planning were used was highly associated with the usage of the gamification and the chatbot. The interrelationship between usage of the progress graphs and information was higher than their association with the rest of the intervention. The number of days the Fitbit was used was independent from the overall app use frequency.

#### Exploring Determinants of Engagement

**Figure 3.**
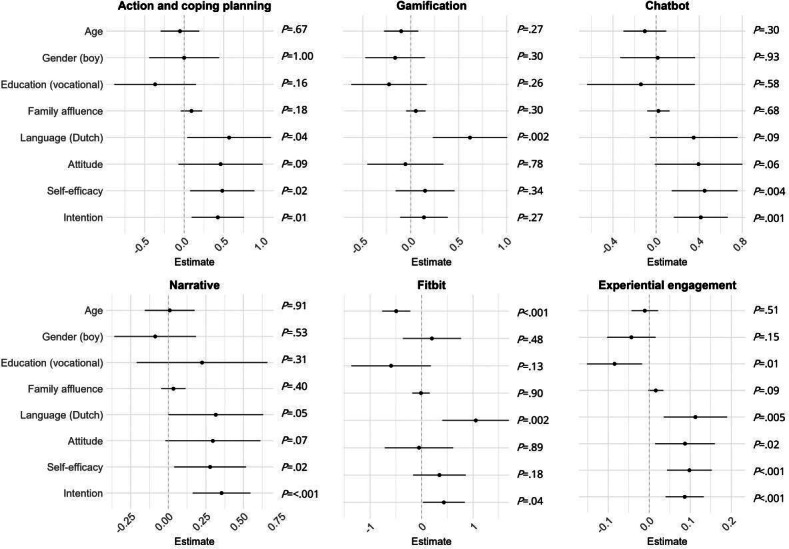
Exploratory analysis of participant characteristics predicting engagement indicators using univariate linear mixed models (1 predictor per model) with school class as a random intercept (N=159). Frequency outcomes were log-transformed. Estimates shown with 95% CIs and *P* values.

For each participant characteristic and baseline behavioral determinant, we ran a separate univariate linear mixed model including school class as random intercept to predict the engagement indicators. Results are shown in Figure 3, showing the estimates with 95% CIs and corresponding *P* values. Descriptive statistics for the engagement indicators across levels of the different determinants are shown in Multimedia Appendix 4. Fitbit usage varied as a function of age, with older adolescents using the Fitbit for fewer days. Age did not influence the other engagement indicators. Participants who did not speak Dutch as their primary language at home exhibited lower engagement with all components of the intervention. Gender, education type, and family affluence did not predict engagement. A positive attitude toward a healthier lifestyle, self-efficacy to change health behaviors, and the intention to change health behaviors were associated with higher usage of the action and coping planning, the chatbot, and the narrative components. However, for the gamification and Fitbit components, attitude and self-efficacy did not predict usage and intention to a lesser extent. Regarding experiential engagement with the app, participants in the general or technical education track, as well as those who spoke Dutch as their primary language at home, provided more positive experiential evaluations than participants in the vocational track or those speaking another maternal language. Additionally, higher attitude, self-efficacy, and intention were linked to more positive reporting of experiential engagement.

### Identifying Engagement Styles

**Figure 4.**
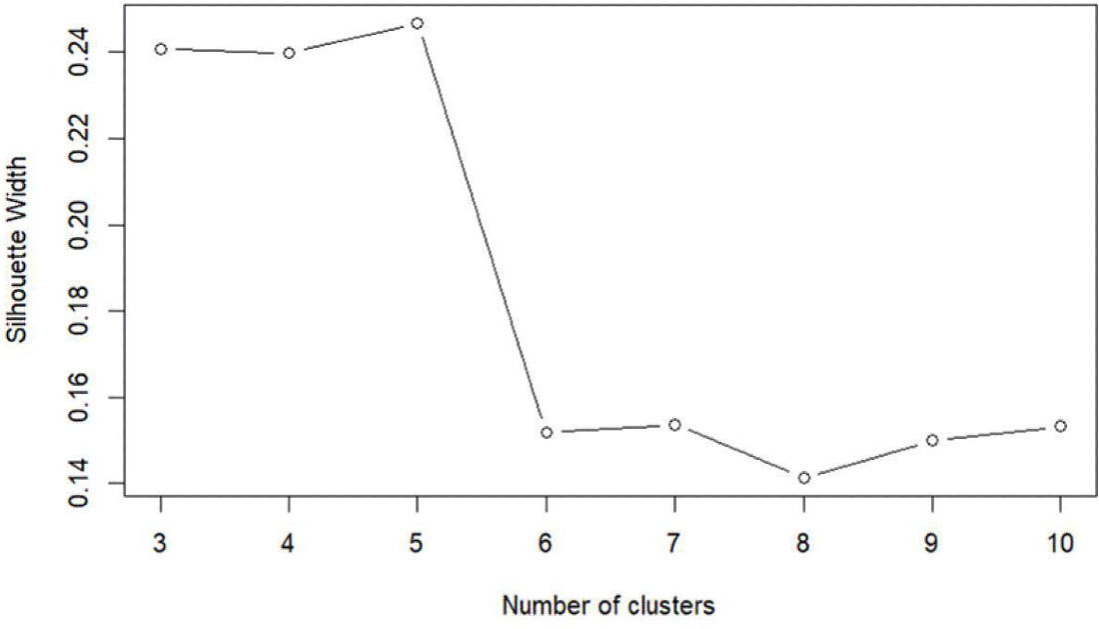
Average silhouette width for different numbers of clusters.

Cluster solutions with 3, 4, or 5 clusters had comparable average silhouette width (Figure 4). Exploring these cluster solutions, it was observed that different clusters were formed based on the extent to which the Fitbit was used, the narrative was watched, and the rest of the app was used. There was no clear separation between clusters according to the extent of usage of the chatbot. Because a 5-cluster solution had the greatest silhouette width but had 1 cluster that was considered too small for follow-up analyses (n=9), the 4-cluster solution was chosen.

**Figure 5.**
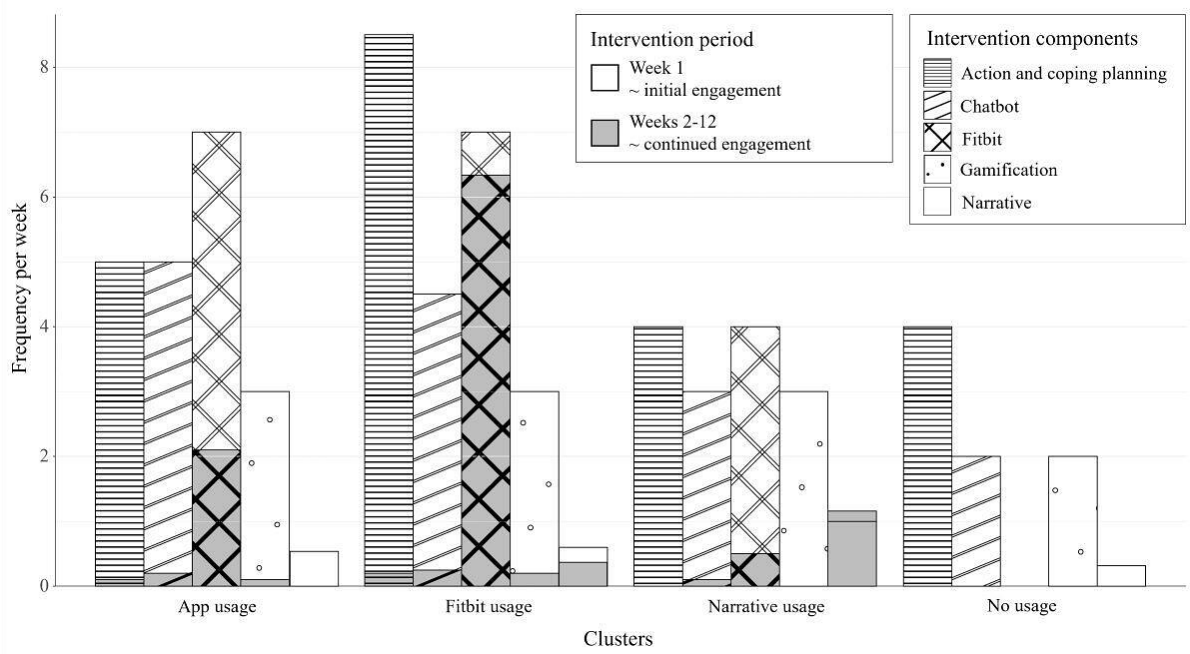
Average usage of the main intervention components per engagement style. Usage is presented separately for week 1 (white bars) and the weekly average of weeks 2-12 (gray bars). Presented are the median of the frequency per week using the component or the days per week wearing the Fitbit. For the narrative, the mean was calculated on the self-reported number of watched episodes after week 1 (0=none, 1=1, or 2=all 2) and after week 12 (0=less than 3, 1=3-11, and 2=all 12).

Median usage of the intervention components per cluster is shown in Figure 5. The 4 clusters were labeled according to what distinguishes them from each other. Participants classified in the “narrative usage” cluster (n=19) were characterized by having watched multiple episodes of the narrative, those in the “Fitbit usage” cluster (n=32) by a relatively high and sustained usage of the Fitbit, those in the “app usage” cluster (n=36) had used the intervention but with less explicit usage of the Fitbit or narrative, and participants classified in the “no usage” cluster (n=72) had overall low to no usage of the intervention.

### Determinants of Engagement Style

Statistics of the final multinomial logistic regression model are shown in Multimedia Appendix 2 and the statistically significant associations are reported here. Younger adolescents were more likely to be in the Fitbit usage group than in the no usage group (odds ratio [OR] 0.30, 95% CI 0.14‐0.65; *P*=.002). Participants who reported lower peer support were more likely to be in the app usage group than in the no usage group (OR 0.43, 95% CI 0.25‐0.74; *P*=.002) and more likely to be in the Fitbit usage group than in the app usage group (OR 1.96, 95% CI 1.08‐3.55; *P*=.03). Participants who reported more depressed feelings were more likely to be in the no usage group than in the app usage group (OR 0.33, 95% CI 0.17‐0.66; *P*=.002). No other baseline characteristics were significant predictors of engagement cluster.

### Predictive Value of Engagement Style

The changes in the health behaviors from preintervention to postintervention did not differ significantly between clusters (Table 4).

**Table 4.** Effects of engagement cluster on preintervention to postintervention change in health behaviors^a^.

	N	Chi-square (*df*)	*P* value
Physical activity, ENMO^b^ (mg)	147	1.7 (3)	.63
Sedentary time (minutes)	148	0.9 (3)	.82
Sleep regularity (minutes)	132	1.5 (3)	.68
Sleep quality (scale 1‐5)	159	2.0 (3)	.57
Breakfast (days per week)	159	4.6 (3)	.21

a Linear mixed models with subject and school class as random intercepts.

b ENMO: Euclidean Norm Minus One.

## Discussion

### Differential Engagement Between Components Over Time

This study investigated engagement with the multicomponent mHealth intervention #LIFEGOALS. #LIFEGOALS consisted of an app coupled with an activity tracker and included self-regulation guidance, gamification elements, health information, a support chatbot, and narrative videos. We aimed to gain insight into how the intervention is used, how components relate to each other, which users apply which engagement style, and how engagement style relates to health outcomes. The combination of system usage and questionnaire data allowed for the investigation of both behavioral and experiential engagement.

On average, users rated their initial experience when using the app as positive. The components that were used the most were the action and coping planning and the Fitbit, followed by the gamification and the chatbot. In the first week, many users used nearly all the intervention components (ie, self-regulation techniques, gamification, chatbot, and Fitbit). However, overall usage dropped steeply after the first week. The decrease in usage was least observed in the number of days wearing the Fitbit, although the use of the Fitbit decreased over time. This is in line with previous research showing that providing a Fitbit is not enough to achieve sustained usage [44]. It may well be that initial engagement is exploratory in nature, given that for all components, median usage was the highest in the first week, after which the extent of usage decreased. The rating of experiential engagement (ie, interest, affect, attention, and usefulness) slightly decreased only after the first week, potentially indicating that mHealth acceptability does not predict sustained mHealth usage. However, common self-reported reasons for low usage of the #LIFEGOALS intervention were low ease of use, technical problems, next to lack of time, forgetting, or no interest in the app. These findings confirm previous research highlighting the importance of technical quality and ease of use for mHealth engagement [45,46]. This might particularly play a role for adolescents in the vocational education track. They, along with participants whose maternal language was not Dutch, rated their experience while using the app less positively than participants in the general or technical education track. Another paper on this trial, moreover, found adolescents in the vocational education track to be more likely to drop out from using the intervention [13]. Overall, health is not a main concern for many adolescents, and vocational students may be less motivated to change health behaviors [13]. During data collection, it was indeed observed that vocational students showed lower motivation to participate in the study and to use the app than participants in the general track. A lower education level and Dutch language proficiency may have also contributed to greater difficulties in using the app. One proposed strategy for tailoring interventions to individuals with lower education attainment is to provide a support person with whom they can have direct contact [13].

Furthermore, exploration of engagement with #LIFEGOALS provided insight into the popularity and interconnectedness of specific intervention components. Engagement was the highest with the intervention strategies intended to enhance *self-regulation*. That is, action and coping planning were visited the most in the app, and sustained usage was the highest with the self-monitoring device (ie, the Fitbit). These components are theoretically considered important for behavior change, as has been shown in other studies [47,48]. Furthermore, usage of the action and coping planning was moderately to highly associated with usage of the *gamification*, suggesting that the gamification succeeded in its role to motivate users to set and complete action plans. Especially for an adolescent population, gamification has been put forward as an essential element within mHealth [46,49]. Results suggest that a points system with avatar appears to be an effective combination of gamification techniques. Notably, baseline attitude toward a healthy lifestyle and intentions to change health behaviors were not associated with subsequent use of the gamification features or the Fitbit. However, they did influence engagement with other components of the intervention. This pattern may suggest that reward-based elements, such as gamification and Fitbit features, have the potential to engage adolescents with lower behavioral motivation. In contrast, components such as the chatbot and narrative, which could also be considered motivational, may have less potential in this regard. There was a particularly low usage of the narrative video episodes, as well as with the tips with information for healthy lifestyles. In #LIFEGOALS, the gamification features were not designed to stimulate engagement with the health narrative or information in the app, which could partly explain the low usage of these elements. Postintervention interviews with users suggested that tailored advertisement could improve engagement with the narrative videos [22]. Future research is needed to formulate an answer to whether increased embeddedness in the app, linkage with gamification, or other techniques could push engagement with health narrative video episodes as part of mHealth.

### Engagement Styles

Cluster analyses using multiple indicators of engagement classified users according to the extent of overall usage and to the extent of usage of different intervention components. Besides a no usage cluster, the remaining clusters were characterized by relatively high usage of the health narrative, high usage of the Fitbit, or no explicit usage of the narrative or Fitbit. The identification of different clusters depending on the extent of usage of a certain intervention component was also found in other studies investigating clusters of engagement with a multicomponent intervention [17,20]. Experiential engagement did not influence the clustering in this study. It might be that experiential engagement is reflected in the amount of usage (considering, eg, that the experience of flow implies a loss of track of time, which will translate in longer in-app duration), thus explaining little additional variance on which to base cluster grouping. Indeed, previous research has indicated significant correlations between self-reported experiential engagement and logged behavioral engagement [28]. In what follows, we discuss the 4 engagement styles that were distinguished in this study, together with their determinants.

A small group of users (19/159, 12%) was classified with a *narrative usage* style, characterized by the relatively high number of health narrative episodes watched. They all reported having watched 3 or more (out of 12) episodes, did not consistently wear the Fitbit, and had average usage of the self-regulation techniques and chatbot. Based on theories on narrative persuasion, we expected that adolescents with low motivation and ability to cognitively elaborate on how to live healthier would more easily be persuaded by a health message embedded in a narrative [26]. We found no evidence that low motivation or low ability increased the likelihood of watching the narrative: low intention to live healthier (as a proxy for motivation) or vocational education type (indicative of lower ability for cognitive processing) did not increase the odds of being in the narrative usage cluster. In contrast, exploratory linear regressions showed greater narrative usage among participants with higher intention. It could be that motivation and ability do not predict who will watch the narrative but rather who would be more easily influenced by watching the narrative. Future studies with a larger sample size are needed to investigate this question.

The group of users classified with a *Fitbit usage* engagement style (32/159, 20% of the participants) wore the Fitbit nearly every day of the 12 weeks. Their usage of the app ranged from a little exploration of some components to high initial and sustained engagement with the entire app. The probability of engaging with a Fitbit usage style compared with a no usage style decreased with age (OR=0.30). Evidence indicates that although adolescents perceive the Fitbit as easy to use, they also consider it as effort-demanding [44,50]. It could be that young adolescents are more easily entertained by the Fitbit features, hence outweighing the effort required to use the Fitbit. Participants who reported higher perceived peer support were 96% more likely (OR=1.96) to continuously use the Fitbit (Fitbit usage style) than the app components (app usage style). It could be that supportive peers encouraged usage of the Fitbit by providing the opportunity to compare progress and achievements or to use the device together. Indeed, social support has been found to facilitate positive change in physical activity [51,52].

A quarter of the participants (36/159, 23%) showed usage of most or all the intervention components and were categorized in the *app usage* engagement style. The probability of engaging with an app usage style compared with a no usage style differed according to peer support and depressed feelings. Adolescents who perceived higher peer support were less likely to engage with an app usage style than with a no usage style (OR=0.42) or a Fitbit usage style (OR=0.51). It could be that the experience of sufficient support in one’s social network makes a health app seem redundant. Also, participants reporting more depressed feelings were less likely to engage with an app usage style than with a no usage style (OR=0.34). Individuals with a low mood may lack the energy required to engage with the app and have been found to engage less with mHealth also in other studies [45].

The largest cluster was the *no usage* cluster, consisting of 45% (72/159) of the participants who had not or rarely used any component of the intervention after the first week. Previous studies analyzing clusters of engagement have also identified 1 group with very low to no usage [15-17,20]. Not using the intervention should not cause concern if this reflects that the individual already meets the objectives of the intervention [53]. Individuals who already perform the behaviors that are targeted by the mHealth intervention, for instance, may perceive lower usefulness and use the intervention to a lesser extent than individuals who are aware that they could make important health behavior improvements. Baseline descriptive statistics indeed indicated that a part of the sample already maintained a healthy lifestyle. However, healthier behaviors at baseline did not significantly increase the odds of a no usage engagement style, but this might be due to low statistical power given the many missing accelerometry data and relatively small sample sizes of the clusters. It is an interesting avenue to further investigate baseline levels of health behaviors as predictors of engagement style, as this could also provide an answer to the question whether the intervention reaches the target group with the highest need.

### Effective Engagement

To gain insight into the characteristics of engagement that are most effective for achieving intervention effects, we analyzed whether health behavior change differed according to engagement style. Our results did not show an effect of engagement style on change in physical activity, sedentary behavior, sleep duration, sleep quality, or frequency of breakfast consumption. Among others, the overall low engagement with #LIFEGOALS may have complicated the detection of differences in health effects between engagement styles, given that also the clusters other than the no usage cluster contained many participants with little overall usage and low continued use. Low engagement, however, does not necessarily imply a failure of the intervention to reach health effects. Usage of the intervention components (ie, behavioral engagement) and the experience while using the intervention (ie, experiential engagement) should lead to engagement with the behavior change processes that are related to the intervention goals [54]. A decrease in engagement with the intervention may then be a positive phenomenon if this reflects continued engagement with the behavioral processes. This could provide an explanation of why there exists inconsistency on whether more sustained engagement is related to greater intervention effects [15,55]. To understand “effective engagement,” more studies on different mHealth contexts are needed to clarify the type and amount of engagement that are minimum required for reaching health effects.

### Limitations

System usage data are a complex entity, introducing challenges to adequately register and extract engagement indicators. In our study, all registered activity in the app or with the activity tracker was assigned to the participant who logged in on these devices during installation, for which we cannot rule out that these logged data may have included activity of other individuals using those devices. Furthermore, for the duration spent in the app, we detected an error for defining the end of a session (ie, in specific circumstances, no end duration was logged). Therefore, frequency data were used instead. There are multiple ways to operationalize behavioral engagement, and we could have also opted for the intensity of use (eg, the rate of visited pages or the number of action plans created) [56]. Because different indicators of behavioral engagement might reveal different effects [18], the multiple ways to operationalize the usage of mHealth components make it difficult to compare log usage data between studies. Next, experiential engagement was measured by self-report ratings of interest, affect, attention, and usefulness. It might be that this measure did not adequately reflect individual variance in the concepts it aims to measure, which could be why experiential engagement did not influence clustering into engagement styles in this study. Future research could consider alternative measures of experiential engagement, such as ecological momentary assessment or questionnaires with more tangible items (eg, ease of use and satisfaction as in the USE Questionnaire [57]) rather than relying on more abstract items such as “I was focused when I used the app,” which may be difficult for young adolescents to self-report retrospectively. Additional research is needed to investigate how best to measure the user experience when interacting with the technology and its additional value for understanding mHealth engagement. Furthermore, the data collection took place during the COVID-19 pandemic, but for the behavior change predictions, we did not control for COVID-19–related measures due to the small sizes of the subsamples of engagement styles. Generalization of the findings to a nonpandemic situation should be done with caution, given that the effect study of #LIFEGOALS showed moderation by pandemic-related restrictions for education and sports [22]. Moreover, the elevated missing data caused by using accelerometers to assess activity-related behaviors limited the statistical power to study health behaviors as determinants or outcomes of engagement styles. Overall, the low usage of the intervention limited the possibilities to identify engagement styles and detect differences between styles. A high percentage of nonusage is also observed in other mHealth studies [5,12,58], but several participants mentioned low ease of use, and the intervention might indeed have lacked in-app guidance and tutorials on what to do in the app. The overall look and feel of apps have been found to be an important factor for adolescents to adopt health apps [59], and the design and technical functioning of #LIFEGOALS were likely of lower quality than those of commercial apps. The high technical requirements of end users challenge the possibilities to investigate mHealth engagement in noncommercial research contexts.

### Future Directions and Conclusions

This study was the first, to our knowledge, to focus specifically on user styles of engagement with mHealth among adolescents. Four engagement styles were identified: narrative usage, Fitbit usage, app usage, and no usage. The styles differ in extent of usage and in preferences for certain intervention components. For instance, adolescents with lower perceived social support showed higher engagement with the app, while depressed feelings, consistent with prior research, were inversely associated with mHealth usage. Interestingly, Fitbit usage was independent of app usage, with younger adolescents more likely to use the Fitbit than the older ones. Moreover, our results reaffirm the important role of social support in sustaining engagement with wearable activity trackers [51]. Interventions designed to promote physical activity are likely to benefit from incorporating interactive components to foster continued physical activity.

Furthermore, our findings support previous research emphasizing the importance of technical quality and intuitive design in fostering mHealth engagement [45,46]. These aspects may be particularly crucial for adolescents with lower education levels or from immigration backgrounds, who are already at higher risk for unhealthy behaviors. To avoid leaving behind those who might benefit the most, digital health interventions must ensure optimal navigability and ease of use. For these populations, offering in-person support may further improve accessibility and uptake [13].

Moreover, our results provide support for the inclusion of reward-based elements and self-regulation. The findings from our exploratory analyses suggest that adolescents’ attitudes, self-efficacy, and intentions toward healthy living may influence engagement with mHealth behavior change interventions. In primary prevention, where intrinsic motivation to change behavior may be low, reward-based elements such as gamification and Fitbit features appear especially promising. In our study, engagement with these components did not depend on behavioral motivation, while our findings suggest that reward-based components can engage users to set and complete action plans. In contrast, components such as the chatbot and narrative, although potentially motivational, may be less effective in engaging less motivated users. Self-regulation techniques, previously shown to be effective in promoting behavior change [47,48], were used most frequently in our study, underscoring their value in mobile behavior change interventions. The identified variations in usage of the mHealth intervention across individual characteristics underscore the importance of tailoring mHealth to the needs and motivations of diverse populations in order to ensure accessibility and sustained engagement.

Our results encourage the continuation of upcoming research aiming to tailor mHealth interventions to user profiles. Addressing the challenge of generally low engagement of mHealth remains crucial, as higher usage could provide more robust findings about the type and duration of engagement necessary to exert meaningful health effects. It can then be investigated for whom and under which circumstances a nudging toward a promising engagement style may be both feasible and beneficial.

## Supplementary material

10.2196/59041 Multimedia Appendix 1 Indicators of engagement included in the cluster analyses.

10.2196/59041 Multimedia Appendix 2 Multinomial logistic regressions of determinants of engagement style.

10.2196/59041 Multimedia Appendix 3 Descriptive statistics and correlation matrix of engagement with the different intervention components.

10.2196/59041 Multimedia Appendix 4 Exploring determinants of engagement.

10.2196/59041 Checklist 1 CONSORT e-HEALTH (V1.6.1) checklist.

## Abbreviations

mHealth: mobile health
OR: odds ratio
PROMIS-PedDepSx: Patient-Reported Outcomes Measurement Information System Pediatric Bank Depressive Symptoms v2.0
