# Supplementary material for: Adolescent Engagement With a Multicomponent mHealth Tool: Identifying Usage Patterns, Determinants, and Health Behavior Change in an Intervention Trial
Source: JMIR Mhealth Uhealth. 2025 Aug 18;13:e59041. doi: 10.2196/59041 (PMC12360726; doi:10.2196/59041)
Supplement: Multimedia Appendix 1 [file mhealth-v13-e59041-s001.docx]

## Multimedia Appendix 1. Indicators of engagement included in the cluster analyses

Exploratory cluster analyses were performed to identify different styles of engagement with the #LIFEGOALS intervention. A decision about which indicators of engagement to include in the final cluster analysis was based on the descriptive statistics of the engagement data, and on the theoretical and evidence-based rationale for inclusion of the different components in the intervention. As such, engagement with elements of the intervention that were used very little in general (these were the progress graphs and the information component), were excluded from the cluster analyses. Next, experiential engagement with the narrative and with the other parts of the app were left out of the final cluster analysis because these did not influence the cluster solutions. The action and coping planning components were considered an important aspect of the intervention to obtain behavior change, and thus of interest to differentiate between clusters. Descriptive statistics showed a strong association between action and coping planning usage, which is why they were summed together forming an engagement indicator for the ‘usage of action and coping planning’. Descriptive statistics moreover showed that the frequency of usage of action and coping planning varied over time. For this reason, a time dimension was added to explore whether different clusters could be formed for initial engagement (i.e., first week) versus sustained engagement (i.e., weeks 2 to 6, and weeks 7 to 12) with this component. The number of days using the Fitbit also varied over time and was independent of the usage of other components of the intervention. For that reason, it was added to the cluster analysis with a time dimension: separate variables were created for week 1, weeks 2 to 6, and weeks 7 to 12. However, because cluster solutions in the exploratory analyses did not differentiate between Fitbit usage in week 1 and weeks 2 to 6, these were later taken together. Frequency of usage of the gamification and the chatbot were hypothesized to boost engagement and hence considered valuable to include in the cluster analysis. Because they were used very little after the first week, no time dimension was added. The theoretical rationale of the narrative component was to reach a specific group of adolescents (those with low motivation or ability to consciously elaborate on the health message) and bring about behavior change via a different route of information processing than the other components (i.e., the periphery route), which is why the extent of watching the narrative was added to the cluster analysis. For the number of episodes watched, the answer options ‘zero’ and ‘one or two’ were combined, forming three ordinal categories: ‘zero to two’, ‘three to eleven’ and ‘all twelve’ episodes watched. This was done because in the third wave of data collection, the first episode of the narrative was shown in the classroom. The indicators of engagement included in the final cluster analysis are presented in Text box MA1.

**Text box MA1**. Indicators of engagement included in the cluster analysis

| Indicators of engagement included in the cluster analysis:   - Usage of action and coping planning in week 1 (frequency) |
| --- |
| - Usage of action and coping planning in weeks 2 to 6 (frequency) |
| - Usage of action and coping planning in weeks 7 to 12 (frequency) |
| - Usage of the Fitbit in weeks 1 to 6 (days used) |
| - Usage of the Fitbit in weeks 7 to 12 (days used) |
| - Usage of gamification (12-week frequency) |
| - Usage of the chatbot (12-week frequency) |
| - Number of episodes of the narrative watched (self-reported: ‘0 to 2’, ‘3 to 11’, or ‘all 12’) |
