# Supplementary material for: Adolescent Engagement With a Multicomponent mHealth Tool: Identifying Usage Patterns, Determinants, and Health Behavior Change in an Intervention Trial
Source: JMIR Mhealth Uhealth. 2025 Aug 18;13:e59041. doi: 10.2196/59041 (PMC12360726; doi:10.2196/59041)
Supplement: Multimedia Appendix 2 [file mhealth-v13-e59041-s002.docx]

## Multimedia Appendix 2. Multinomial logistic regressions of determinants of engagement style

Multinomial logistic regressions were fitted with engagement cluster as dependent variable to investigate the associations between the baseline characteristics and clustering. Because of the higher amount of missing accelerometry data, different models were built for the health behaviors (PA, SB, sleep routine, sleep quality and breakfast frequency) (Table MA2.1) and the other characteristics (demographics, behavioral determinants, mental health and education restrictions) (Table MA2.2). Models were adjusted for the COVID-19 related variable Education Restrictions.

The variables that indicated a potential association with clustering were retained and included in the final multinomial logistic regression model. We analyzed the predictive ability of each variable for classification into one specific cluster, relative to each of the other three clusters. All pairwise comparisons are included. The results of these analyses are summarized in Table MA2.3.

**Table MA2.1** Multinomial logistic regressions predicting engagement style (N=158).

|  | *χ^2^* | *df* | *p* |
| --- | --- | --- | --- |
| PA (ENMO) | 4.21 | 3 | .240 |
| SB | 1.66 | 3 | .647 |
| Sleep routine | 0.36 | 3 | .949 |
| Sleep quality | 0.91 | 3 | .824 |
| Breakfast frequency | 1.51 | 3 | .680 |
| Education restrictions | 0.33 | 3 | .954 |

**Table MA2.2** Multinomial logistic regressions predicting engagement style (N=158).

|  | *χ^2^* | *df* | *p* |
| --- | --- | --- | --- |
| Gender | 2.28 | 3 | .516 |
| Age | 13.69 | 3 | .003* |
| FAS | 11.19 | 6 | .108 |
| HRQoL | 3.31 | 3 | .346 |
| Psychological wellbeing | 0.54 | 3 | .910 |
| Self-Perception | 7.70 | 3 | .053· |
| Moods | 4.45 | 3 | .217 |
| Peer support | 7.43 | 3 | .056· |
| Resilience | 2.37 | 3 | .499 |
| Depressed feelings | 8.80 | 3 | .032* |
| Attitude | 5.27 | 3 | .153 |
| Self-efficacy | 1.34 | 3 | .720 |
| Intention | 7.69 | 3 | .053· |
| Education restrictions | 7.05 | 3 | .070· |

**Table MA2.3** Multinomial logistic regression analyses predicting engagement style (N=158).^a^

|  | Narrative- vs. No-usage | | | Fitbit- vs. No-usage | | |
| --- | --- | --- | --- | --- | --- | --- |
|  | *β* | SE | *P* | *β* | SE | *P* |
| Age | -0.72 | 0.42 | .09 | -1.19 | 0.38 | .002 |
| Intention | 0.28 | 0.30 | .36 | 0.02 | 0.27 | .95 |
| Self-perception | -0.25 | 0.34 | .46 | 0.30 | 0.31 | .32 |
| Peer support | -0.44 | 0.31 | .16 | -0.17 | 0.27 | .52 |
| Depressed feelings | -0.54 | 0.39 | .16 | -0.37 | 0.31 | .23 |
|  | App- vs. No-usage | | | Narrative- vs. App-usage | | |
|  | *β* | SE | *P* | *β* | SE | *P* |
| Age | -0.37 | 0.32 | .25 | -0.34 | 0.45 | .45 |
| Intention | 0.41 | 0.26 | .11 | -0.13 | 0.34 | .70 |
| Self-perception | -0.40 | 0.30 | .18 | 0.15 | 0.38 | .70 |
| Peer support | -0.85 | 0.27 | .002 | 0.41 | 0.34 | .23 |
| Depressed feelings | -1.09 | 0.35 | .002 | 0.55 | 0.45 | .22 |
|  | Fitbit- vs. App-usage | | | Narrative- vs. Fitbit-usage | | |
|  | *β* | SE | *P* | *β* | SE | *P* |
| Age | -0.82 | 0.42 | .05 | 0.47 | 0.49 | .34 |
| Intention | -0.39 | 0.31 | .20 | 0.26 | 0.35 | .46 |
| Self-perception | 0.70 | 0.36 | .05 | -0.56 | 0.40 | .16 |
| Peer support | 0.67 | 0.30 | .03 | -0.27 | 0.34 | .43 |
| Depressed feelings | 0.39 | 0.39 | .06 | -0.18 | 0.43 | .68 |

Models are adjusted for COVID-19 related parttime home education. ^a^ Narrative-usage = cluster characterized by watching multiple episodes of the narrative (n=19); Fitbit-usage = cluster characterized by high usage of the Fitbit (n=32); App-usage = cluster with less explicit usage of the narrative or Fitbit (n=36); No-usage = cluster with overall low to no usage (n=72).
