# Supplementary material for: Adolescent Engagement With a Multicomponent mHealth Tool: Identifying Usage Patterns, Determinants, and Health Behavior Change in an Intervention Trial
Source: JMIR Mhealth Uhealth. 2025 Aug 18;13:e59041. doi: 10.2196/59041 (PMC12360726; doi:10.2196/59041)
Supplement: Multimedia Appendix 3 [file mhealth-v13-e59041-s003.docx]

## Multimedia Appendix 3. Descriptive statistics and correlation matrix of engagement with the different intervention components

The descriptive statistics of the behavioral engagement with the app components and the Fitbit, and of the experiential engagement with the intervention, are presented in boxplots in Figures MA3.1 to MA3.3. Distribution bar plots and a correlation matrix of the usage of the intervention components are presented in Figure MA3.4.


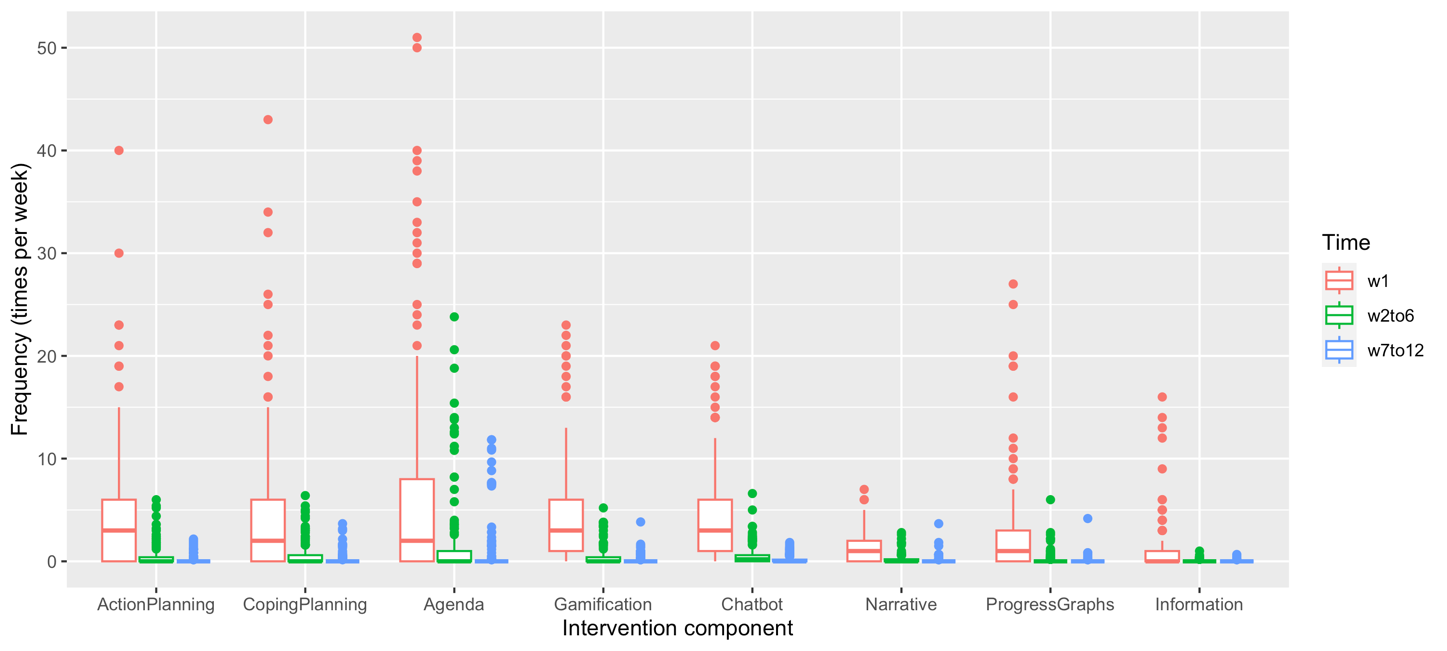

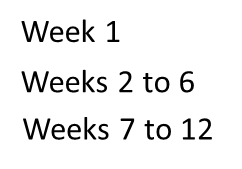


**Figure MA3.1.** Extent of usage of the different app components for week 1, weeks 2 to 6, and weeks 7 to 12.


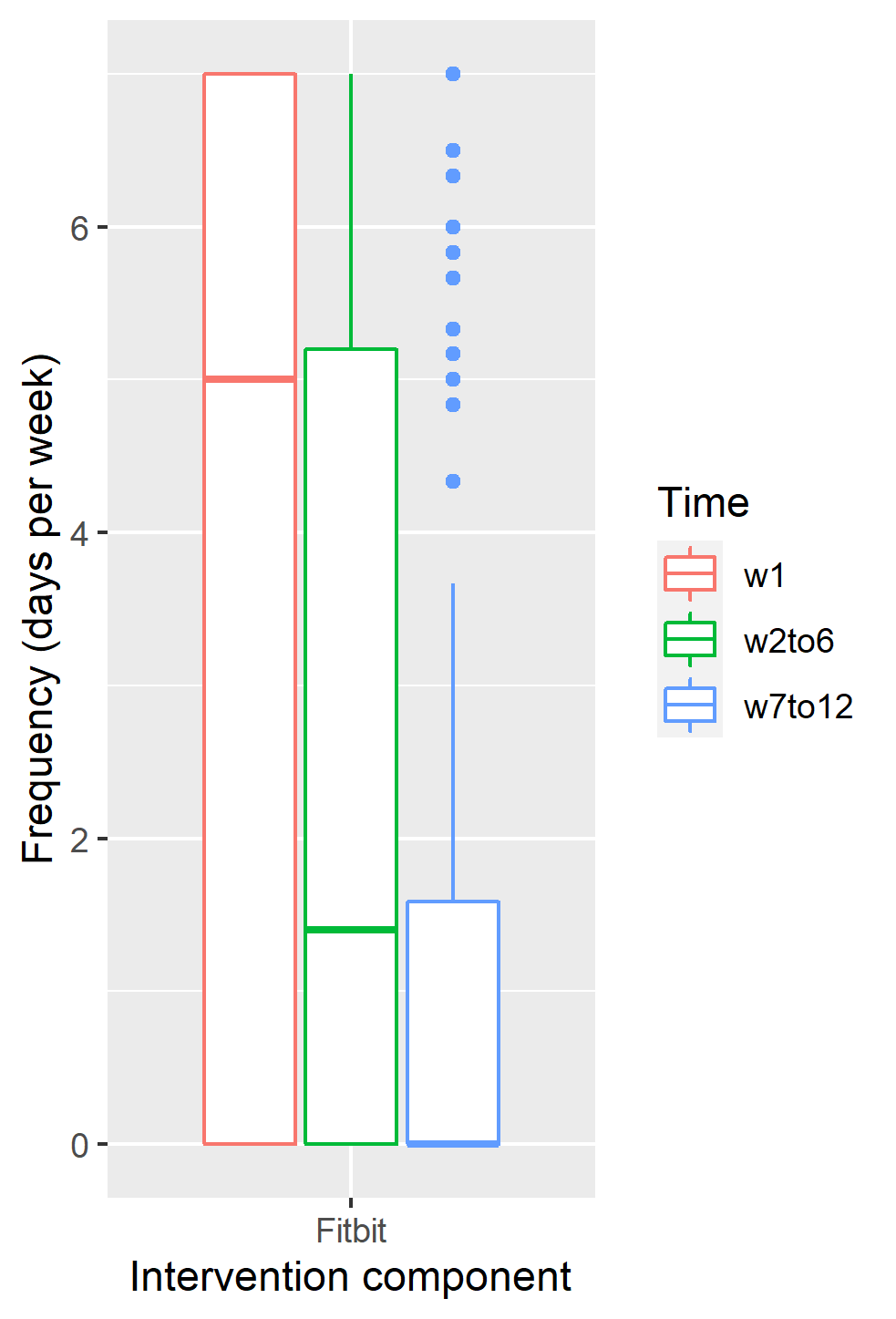

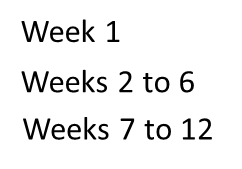


**Figure MA3.2.** Extent of usage of the Fitbit for week 1, weeks 2 to 6, and weeks 7 to 12.


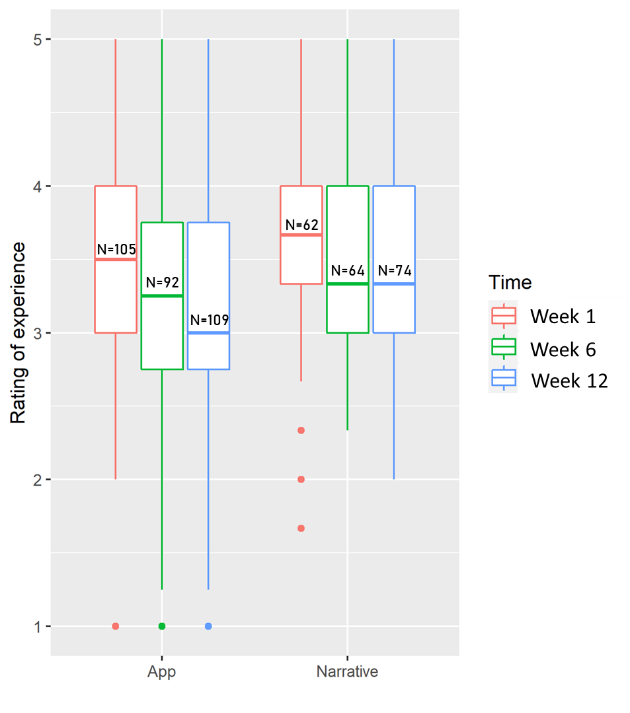


**Figure MA3.3.** User experience with the narrative component (Narrative) or with the other components of the app (App) after week 1, week 6, and week 12. Higher scores indicate more positive experiential engagement.

**
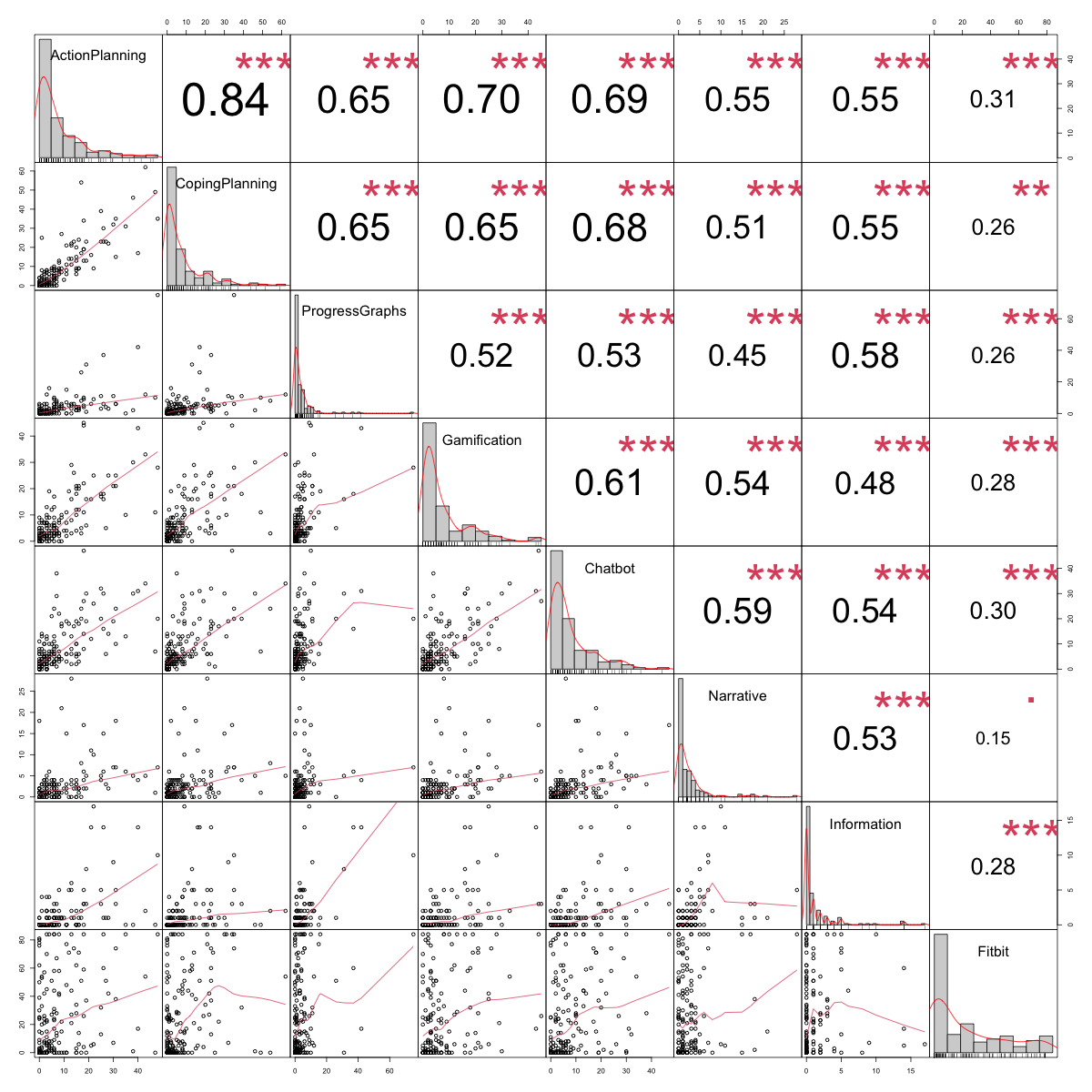
**

**Figure MA3.4.** Spearman correlation matrix of the different app components with distribution bar plots.
