# Supplementary material for: Adolescent Engagement With a Multicomponent mHealth Tool: Identifying Usage Patterns, Determinants, and Health Behavior Change in an Intervention Trial
Source: JMIR Mhealth Uhealth. 2025 Aug 18;13:e59041. doi: 10.2196/59041 (PMC12360726; doi:10.2196/59041)
Supplement: Multimedia Appendix 4 [file mhealth-v13-e59041-s004.docx]

## Multimedia Appendix 4. Exploring determinants of engagement.

Table MA4 shows descriptive statistics of different indicators of engagement with the intervention, both for the total sample and separately for different groups based on sociodemographic characteristics and baseline behavioral intention, attitude and self-efficacy.

**Table MA4.** Mean (SD) of engagement indicators as a function of socio-demographic and motivational characteristics^a^

|  | Experiential engagement with app^b^ (scale 1-5) | Action and coping planning (frequency^c^) | Gamification  (frequency^c^) |
| --- | --- | --- | --- |
| Full sample (n=159) | 3.25 (0.69) | 16.43 (20.73) | 7.58 (8.96) |
| Gender^d^  Girl  Boy | *P*=.17  3.32 (0.66)  3.15 (0.71) | *P*=.99  19.67 (24.12)  13.28 (0.71) | *P*=.30  8.67 (10.24)  6.59 (7.27) |
| Age | *P=*.49 | *P=.*67 | *P=.28* |
| Education type  General or Technical  Vocational | *P=*.04  3.32 (0.61)  3.02 (0.87) | *P=*.19  18.00 (21.40)  11.59 (17.90) | *P=*.28  8.15 (9.43)  5.82 (7.17) |
| Family affluence  Low  Middle  High | *P=*.19  3.10 (0.67)  3.36 (0.73)  3.22 (0.60) | *P=*.43  11.36 (14.97)  17.64 (21.00)  20.57 (25.53) | *P=*.50  5.91 (7.37)  8.62 (10.29)  7.51 (7.54) |
| Language at home  Dutch (n=)  Other (n=) | *P*=.006  3.32 (0.66)  2.87 (0.72) | *P=*.04  18.19 (21.82)  8.83 (12.93) | *P=*.007  8.52 (9.51)  3.53 (4.26) |
| Attitude | *P=.03* | *P=.02* | *P=.18* |
| Low (n=43) | *3.04 (0.54)* | *13.07 (17.22)* | *6.84 (8.86)* |
| High (n=116) | *3.32 (0.72)* | *17.67 (21.83)* | *7.85 (9.02)* |
| Self-efficacy | *P=.018* | *P=.11* | *P=.68* |
| Low (n=121) | *3.17 (0.69)* | *16.07 (21.02)* | *7.35 (8.79)* |
| High (n=38) | *3.46 (0.64)* | *17.55 (20.01)* | *8.32 (9.59)* |
| Intention | *P=.018* | *P=.04* | *P=.10* |
| Low (n=63) | *3.07 (0.61)* | *13.54 (18.66)* | *6.48 (8.38)* |
| High (n=96) | *3.35 (0.71)* | *18.33 (21.87)* | *8.30 (9.30)* |
|  | Chatbot  (frequency^c^) | Narrative  (frequency^c^) | Fitbit usage (frequency in days) |
| Total | 8.29 (8.98) | 2.62 (4.14) | 24.83 (27.83) |
| Gender^d^  Girl  Boy | *P=*.93  9.37 (9.95)  7.25 (7.79) | *P=*.53  3.12 (5.00)  2.12 (2.92) | *P*=.48  25.56 (29.28)  24.72 (26.51) |
| Age | *P=.30* | *P=.91* | *P=.002* |
| Education type  General or Technical  Vocational | *P=* .59  8.51 (8.77)  7.62 (9.69) | *P=*.33  2.73 (4.61)  2.31 (2.10) | *P*=.16  28.07 (29.22)  15.18 (20.69) |
| Family affluence  Low  Middle  High | *P=* .43  6.49 (7.37)  9.92 (10.01)  7.11 (8.11) | *P=*.29  2.26 (4.00)  2.97 (3.94)  2.34 (4.76) | *P*=.98  20.53 (22.85)  25.33 (28.25)  29.26 (32.42) |
| Language at home  Dutch  other | *P=.09*  8.98 (9.51)  5.33 (5.43) | *P=.0495*  2.82 (4.27)  1.77 (3.44) | *P=*.002  27.63 (28.35)  12.62 (21.99) |
| Attitude | *P=.010* | *P=.012* | *P=.52* |
| Low (n=43) | *6.58 (8.84)* | *1.84 (3.45)* | *23.38 (27.73)* |
| High (n=116) | *8.92 (8.99)* | *2.91 (4.34)* | *24.27 (27.55)* |
| Self-efficacy | *P=.07* | *P=.06* | *P=.74* |
| Low (n=121) | *7.94 (9.06)* | *2.40 (3.85)* | *23.39 (26.31)* |
| High (n=38) | *9.39 (8.75)* | *3.34 (4.93)* | *26.11 (31.48)* |
| Intention | *P=.006* | *P=.037* | *P=.26* |
| Low (n=63) | *6.51 (7.46)* | *2.25 (4.03)* | *21.72 (27.27)* |
| High (n=96) | *9.46 (9.71)* | *2.86 (4.21)* | *25.48 (27.71)* |

^a^ For each indicator of engagement, the *P*-value of the test of difference (linear mixes models with school class as random intercept) is presented.

^b^ Experiential engagement with app = average score of self-report after week 1, week 6, and week 12.

^c^ Frequency of use throughout the 12-week intervention period, log-transformed.

^d^ Two participants identified neither as boy or as girl, and are not included in the table.
