# Supplementary material for: Adolescent Engagement With a Multicomponent mHealth Tool: Identifying Usage Patterns, Determinants, and Health Behavior Change in an Intervention Trial
Source: JMIR Mhealth Uhealth. 2025 Aug 18;13:e59041. doi: 10.2196/59041 (PMC12360726; doi:10.2196/59041)
Supplement: Checklist 1 [file mhealth-v13-e59041-s005.pdf]

CONSORT-EHEALTH checklist (V.1.6.1): Information to include when reporting ehealth/mhealth trials

Manuscript: Styles of engagement with a multicomponent mobile health promotion intervention for adolescents

Authors: Carmen Peuters, Ann DeSmet, Laura Maenhout, Greet Cardon, Dries Debeer, and Geert Crombez

Section/Topic	Item No.	CONSORT* Checklist Item	EHEALTH Extensions	Page in submitted manuscript
**TITLE & ABSTRACT**	1a	Identification as a randomized trial in the title	**i)** **Identify the mode of delivery in the title**.<br>**ii)** Mention **non-web-based components or important co-interventions in the title**<br>**iii)** Mention **primary condition or target group in the title**	NA
	1b	Structured summary of trial design, methods, results, and conclusions NPT** extension: Description of experimental treatment, comparator, care providers, centers, and blinding status	Methods (in Abstract):<br>i) Mention **key features/functionalities/components of the intervention and comparator in the abstract**<br>ii) Clarify the **level of human involvement in the abstract**<br>iii) **Open vs. closed, web-based (self-assessment) vs. face-to-face assessments in abstract**<br>iv) **Results in abstract must contain use data**<br>v) **Conclusions/Discussions in abstract for negative trials**	Page 2, Background and objective<br>none<br>Page 2, methods<br>Page 2, in-app time<br>Page 2, low engagement may have limited detection of health effects
**INTRODUCTION** Background and	2a	Scientific background and explanation of rationale	i) Describe the **problem and the type of system/solution** that is object of the study	Page 3

objectives			ii) **Scientific background, rationale**	Pages 3-4
		Specific objectives or hypotheses	*No EHEALTH-specific additions here*	NA, exploratory. Aims at Page 4
**METHODS** Trial design	3a	Description of trial design (such as parallel, factorial) including allocation ratio	*No EHEALTH-specific additions here*	Page 5
	3b	Important changes to methods after trial commencement (such as eligibility criteria), with reasons	i) ***Bug fixes, Downtimes, Content Changes***	Page 6: reference to [22]
Participants	4a	Eligibility criteria for participants	i) ***Computer / Internet literacy***<br>ii) ***Open vs. closed, web-based vs. face-to-face assessments***<br>iii) ***Information given during recruitment***	Page 5<br>Page 5<br>Page 5-6
	4b	Settings and locations where the data were collected	i) *Clearly **report if outcomes were self-assessed through online questionnaires***<br>ii) ***Report how institutional affiliations are displayed** to potential participants*	Pages 7-9<br>Reference to [22]
Interventions	5	The interventions for each group with sufficient details to allow replication, including how and when they were actually	*i)* ***Mention names, credential, affiliations of the developers, sponsors, and owners***<br>*ii)* ***Describe the history/development process***<br>*iii)* ***Revisions and updating***<br>*iv) Provide information on **quality assurance methods***<br>*v)* ***Ensure replicability by publishing the source code, and/or providing screenshots/screen-capture video, and/or providing flowcharts of the algorithms used.***<br>*vi)* ***Digital preservation***	Page 5: Reference to [22]<br>Page 6, reference to [27]<br>Idem<br>Page 6, reference to [27]<br>Page 5: Reference to [22]

			vii) ***Access***<br>*viii)* *Describe* ***mode of delivery, features/functionalities/components of the intervention and comparator, and the theoretical framework***<br>*ix)* ***Describe use parameters***<br>*x)* ***Clarify the level of human involvement***<br>*xi)* ***Report any prompts/reminders used***<br>*xii)* ***Describe any co-interventions***	Page 5: Reference to [22]<br>Page 6: Reference to [27]<br><br>Pages 7-8<br>Page 6<br>Page 6<br>NA
Outcomes	6a	Completely defined pre-specified primary and secondary outcome measures, including how and when they were assessed	*i)* *If outcomes were obtained through* ***online questionnaires, describe if they were validated for online use***<br>*ii)* ***Describe whether and how "use" (including intensity of use/dosage) was defined/measured/monitored*** *(logins, logfile analysis, etc.).*<br>*iii)* *Describe* ***whether, how, and when qualitative feedback was obtained from participants***	Pages 8-9, reference to [22]<br><br>Pages 7-8<br><br>Described elsewhere [22]
	6b	Any changes to trial outcomes after the trial commenced, with reasons	*No EHEALTH-specific additions here*	Page 6 and more detail in [22]
Sample size	7a	How sample size was determined<br>NPT: When applicable, details of whether and how the clustering by care provides or centers was addressed	*i)* ***Describe whether and how expected attrition was taken into account when calculating the sample size***	This paper concerns secondary analyses, sample size in [22]
	7b	When applicable, explanation of	*No EHEALTH-specific additions here*	NA

		any interim analyses and stopping guidelines		
Randomisation: Sequence generation	8a	Method used to generate the random allocation sequence<br>NPT: When applicable, how care providers were allocated to each trial group	*No EHEALTH-specific additions here*	NA
	8b	Type of randomisation; details of any restriction (such as blocking and block size)	*No EHEALTH-specific additions here*	NA
Allocation concealment mechanism	9	Mechanism used to implement the random allocation sequence (such as sequentially numbered containers), describing any steps taken to conceal the sequence	*No EHEALTH-specific additions here*	NA
Implementation	10	Who generated the random allocation sequence, who enrolled participants, and who assigned participants to interventions	*No EHEALTH-specific additions here*	NA
Blinding	11a	If done, who was blinded after assignment to interventions (for example, participants, care providers, those	*i)* ***Specify who was blinded, and who wasn't***<br>*ii) Informed consent procedures (4a-ii) can create biases and certain expectations -* ***discuss***	NA

		assessing outcomes) and how<br>NPT: Whether or not administering co-interventions<br>were blinded to group assignment		
	11b	If relevant, description of the similarity of interventions	*this item is usually not relevant for ehealth trials as it refers to similarity of a placebo or sham intervention to a active medication/intervention)*	NA
Statistical Methods	12a	Statistical methods used to compare groups for primary and secondary outcomes<br>NPT: When applicable, details of whether and how the clustering<br>by care providers or centers was addressed	*i)* ***Imputation techniques to deal with attrition / missing values***	Pages 10-11
	12b	Methods for additional analyses, such as subgroup analyses and adjusted analyses	*No EHEALTH-specific additions here*	Pages 10-11
Ethics & Informed Consent	X26	*not a CONSORT item)*	*i)* ***Comment on ethics committee approval***<br>*ii)* ***Outline informed consent procedures***<br>*iii)* ***Safety and security procedures***	Page 6, ethical considerations
**RESULTS**<br>Participant flow (a diagram is strongly recommended)	13a	For each group, the numbers of participants who were randomly assigned, received intended treatment, and were analysed for the primary outcome	*No EHEALTH-specific additions here*	Page 5

	13b	For each group, losses and exclusions after randomisation, together with reasons	i) Strongly recommended: An **attrition diagram**	Page 5
Recruitment	14a	Dates defining the periods of recruitment and follow-up	i) **Indicate if critical "secular events" [1] fell into the study period**	Page 5
	14b	Why the trial ended or was stopped [early]	*No EHEALTH-specific additions here*	NA
Baseline data	15	A table showing baseline demographic and clinical characteristics for each group<br>NPT: When applicable, a description of care providers (case volume, qualification, expertise, etc.) and centers (volume) in each group	i) In ehealth trials it is particularly important to **report demographics associated with digital divide issues**,	Page 11
Numbers analysed	16	For each group, number of participants (denominator) included in each analysis and whether the analysis was by original assigned groups	*i)* **Report multiple "denominators" and provide definitions**<br>*ii)* **Primary analysis should be intent-to-treat**	Done<br><br>NA
Outcomes and estimation	17a	For each primary and secondary outcome, results for each group, and the estimated effect size and its precision (such as 95% confidence interval)	*i)* In addition to primary/secondary (clinical) outcomes, the **presentation of process outcomes such as metrics of use and intensity of use** (dose, exposure) and their operational definitions is critical	Presented in Results

Outcomes and estimation	17b	For binary outcomes, presentation of both absolute and relative effect sizes is recommended	*No EHEALTH-specific additions here*	NA
Ancillary analyses	18	Results of any other analyses performed, including subgroup analyses and adjusted analyses, distinguishing pre-specified from exploratory	*i)* A **subgroup analysis of comparing only users** is not uncommon in ehealth trials, but if done it must be stressed that this is a self-selected sample and no longer an unbiased sample from a randomized trial (see 16-iii).	Done
Harms	19	All important harms or unintended effects in each group (for specific guidance see CONSORT for harms)	i) **Include privacy breaches, technical problems**<br>ii) **Include qualitative feedback from participants or observations from staff/researchers**	None to declare<br><br>In Discussion and referred to [22] and [27]
**DISCUSSION** Limitations	20	Trial limitations, addressing sources of potential bias, imprecision, and, if relevant, multiplicity of analyses	**I) Typical limitations in ehealth trials**	Pages 20-21
Generalisability	21		i) **Generalizability to other populations**<br>ii) **Discuss if there were elements in the RCT that would be different in a routine application setting**	Page 21
Interpretation/ Principal Findings	22	Interpretation consistent with results, balancing benefits and harms, and considering other	*i)* **Restate study questions and summarize the answers suggested by the data, starting with primary outcomes and process outcomes (use).**	Page 16

		relevant evidence<br>NPT: In addition, take into account the choice of the comparator, lack of or partial blinding, and unequal expertise of care providers or centers in each group	*ii)* **Highlight unanswered new questions, suggest future research**	Pages 16-22 with future directions at pages 21-22
**OTHER INFORMATION**<br>Registration	23	Registration number and name of trial registry	*No EHEALTH-specific additions here*	Abstract
Protocol	24	Where the full trial protocol can be accessed, if available	*No EHEALTH-specific additions here*	In ref [22]
Funding	25	Sources of funding and other support (such as supply of drugs),<br>role of funders	*No EHEALTH-specific additions here*	Page 22
Competing interests	X27	*(not a CONSORT item)*	i) In addition to the usual declaration of interests (financial or otherwise), also **state the "relation of the study team towards the system being evaluated"**	No conflicts of interest, page 23
